# Nimotuzumab for Patients With Inoperable Cancer of the Head and Neck

**DOI:** 10.3389/fonc.2020.00817

**Published:** 2020-05-27

**Authors:** Tania Crombet Ramos, Braulio Mestre Fernández, Zaima Mazorra Herrera, Normando E. Iznaga Escobar

**Affiliations:** ^1^Center of Molecular Immunology, Havana, Cuba; ^2^Clinical Oncology, National Institute of Oncology and Radiobiology, Havana, Cuba; ^3^Biocubafarma, Havana, Cuba

**Keywords:** nimotuzumab, EGFR, monoclonal antibody, head and neck, radiotherapy

## Abstract

EGFR activation induces cell proliferation, neoformation of blood vessels, survival, and metastasis of the cancer cells. Nimotuzumab is an engineered, intermediate affinity anti-EGFR antibody, that apart from other drugs in its class, is very safe and does not cause hypomagnesemia or grade 3–4 cutaneous rash. The antibody inhibits cell proliferation and angiogenesis, activates natural killer cells, stimulates dendritic cell maturation, and induces cytotoxic T cells. Nimotuzumab restores MHC-I expression on tumor cells, hindering one of the EGFR immune-escape ways. The antibody has been extensively studied in 7 clinical trials, concurrently with irradiation or irradiation plus chemotherapy in subjects with inoperable head and neck tumors. Nimotuzumab was safe and efficacious in unfit patients receiving irradiation alone and in subjects treated with cisplatin and radiotherapy. In patients with locally advanced squamous cell carcinomas of the head and neck, nimotuzumab in combination with low dose cisplatin and radiotherapy was superior to cisplatin and radiotherapy in progression free survival, disease free survival, and locoregional tumor control.

## Introduction

Worldwide, squamous cell carcinomas of the head and neck (SCCHN) constitute the sixth in frequency and the ninth cause of death by cancer (1). The classic risk factors associated with the development of the disease are tobacco and alcohol consumption (2). Experts have estimated that the cancer risk in smokers is 3–15 times higher with respect to non-smokers and it is directly related to the duration of use and the onset of consumption (3). Globally, consumption of alcohol and tobacco causes nearly 65% of SCCHN (4, 5). Recently, viral infections with Epstein-Barr and human papilloma virus (HPV), have also been associated with the occurrence of head and neck neoplasms (6–8).

Surgery and radiotherapy (RT) are the classic therapeutic weapons employed in treating SCCHN (9, 10). Overall, both treatment modalities can be considered equally effective for small tumors. Larger tumors usually require the combined use of both treatment options (11). Curative-intent therapy of stage III or IV SCCHN patients requires a multimodal approach. For inoperable tumors, the preferred alternative is chemo-radiotherapy or radiotherapy plus monoclonal antibodies (MAbs) (12, 13).

Despite the well-established effect in some patients, combined therapy induces adverse events like dermatitis, mucositis, and dysphagia. Treatment is also associated with hematologic toxicity, which augments the threat of hemorrhage or infection (12, 13). After therapy, quality of life is usually impaired because of late complications including sensorineural hearing loss, polyneuropathy, permanent xerostomia, and dysgeusia (14).

## EGFR in Tumorigenesis

Epidermal Growth Factor Receptor type I receptor (EGFR or HER 1) is an oncogene, member of the ErbB/HER family, with tyrosine-kinase activity in the intracellular domain (15, 16). The activation of the EGFR transduces a signal involving cell proliferation, inhibition of apoptosis, angiogenesis, and metastasis (16). EGFR overexpression is detected in ~90% of the SCCHN (17, 18). A recent multivariate analysis demonstrated that overexpression of EGFR in subjects with stage II–IV head and neck neoplasms was associated with early relapses, lower disease-free, and overall survival (19, 20). Two types of molecules, small tyrosine-kinase inhibitors (STKI) and antibodies, have been developed to prevent EGFR signaling (20). Anti-EGFR MAbs recognize the extracellular region of the receptor, preventing activation by ligands (20, 21). Tumor cells destruction can also be mediated by antibody-dependent cell cytotoxicity (ADCC) or complement dependent-cytotoxicity (CDC) (22, 23). Alternatively, STKI bind to the intracytoplasmic domain of HER1, inhibiting its phosphorylation (23, 24).

## Nimotuzumab Mechanism of Action

Nimotuzumab is a humanized anti-EGFR MAb, which was obtained in 1996 after genetic modification of the parental murine molecule ior egf/r3 (25, 26). Nimotuzumab inhibits EGFR phosphorylation in several cell lines overexpressing this oncogene (27–34). The antibody has demonstrated significant antiproliferative, antiangiogenic, and pro-apoptotic activity *in vitro* and in tumor-engrafted mice (32).

In a glioma model, the antibody enhanced the radio-sensitivity by reducing the cancer stem cells (33). Recently, Mazorra and coworkers demonstrated that nimotuzumab activates natural killer (NK) cells, stimulates dendritic cell maturation and induces tumor-recognizing cytotoxic T-cells (34). Besides, the antibody restores MHC-I expression on tumor cells, hindering one of the EGFR immune-escape ways (35, 36).

**Figure 1:**
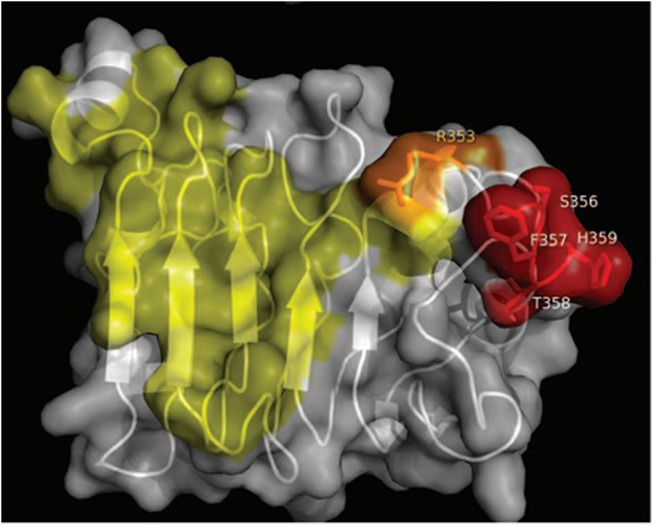
Relative recognition sites of nimotuzumab and cetuximab at domain III of the EGFR. Residues recognized by cetuximab Fab fragment are colored in yellow, while residues recognized by nimotuzumab are colored in red. Overlapping recognition between nimotuzumab and cetuximab is colored in orange (figure reproduced with permissions of the authors).

Nimotuzumab recognition site at the extracellular domain of the EGFR was established by using phage display technology together with extensive mutagenesis of the EGFR and the Fab fragment of the antibody (37). The functional nimotuzumab epitope comprised a stretch of contiguous amino acids (S356-H359) and a non-contiguous residue (R353). Then, nimotuzumab interacting site was compared with the binding place of cetuximab, an approved antibody for the treatment of advanced head and neck cancer patients. The involvement of R353 located within the cetuximab structural epitope indicated some degree of overlapping between both epitopes and explains competition between the antibodies (37). Figure 1 shows the relative recognition sites of nimotuzumab and cetuximab at domain III of the EGFR.

According to a mathematical model developed by our group, antibodies with intermediate affinities like nimotuzumab, would have preferential accumulation in tumors with higher EGFR expression with respect to normal tissues (38). The mathematical model was composed of 4 differential equations reflecting the behavior of the antibody in 4 compartments (plasma, tumor, liver, and skin). Concentration-curves were obtained for each tissue by integrating the differential equations in time; the AUC was obtained by integrating equations again. AUC was assumed as a surrogate of the pharmacodynamic effect of the antibody in the referred tissue (38). The model explained that antibodies with higher affinity would recognize tumors with lower target expression, but also normal tissues (38). Several recent reports give support to this hypothesis (39–41). Akashi and cols. showed that binding of nimotuzumab and subsequent inhibition of the EGFR phosphorylation was detected only in tumor cell lines with medium or high EGFR expression (10^4^ receptors per cell or higher) (39). Furthermore, according to Garrido et al. binding of nimotuzumab Fab fragments was detected only in the A431 cell line (10^6^ receptors per cell), whereas cetuximab Fab fragments also bound to tumor cells with lower EGFR levels (40). As a consequence, higher affinity antibodies preserve efficacy even in those tumors with lower EGFR expression. These antibodies would also be more toxic, inducing on-target off-tumor toxicity such as skin rash and hypomagnesemia (42). For nimotuzumab, patients with high EGFR expression or gene amplification would have a larger benefit. Apart from the previous evidences in the preclinical (39–41) and clinical setting (43–52) on the correlation between EGFR expression and efficacy, this predictive biomarker should be further validated in prospective clinical trials. Table 1 compares the most important characteristic of nimotuzumab vs. cetuximab and panitumumb (37, 41, 53) other marketing approved anti-EGFR MAbs for advanced head and neck and colorectal cancer.

**Table 1:** Comparison of nimotuzumab, cetuximab, and panitumumab on the main characteristics determining their biologic activity.

**Monoclonal antibody**	**Type**	**IgG subclass**	**Immune functions (ADCC)**	**Affinity (EGFR)**	**EGFR binding site in the EGF pocket**
Cetuximab	Chimeric	IgG1	Yes	KD = 10^−10^	D355, Q408, H409, K443, S468
Panitumumab	Fully human	IgG2	No	KD = 10^−11^	D355, K443
Nimotuzumab	Humanized	IgG1	Yes	KD = 10^−9^	R353, S356, F357, T358, H359

## Early Clinical Trials

Since 1998, more than 50 clinical trials have been concluded worldwide with nimotuzumab. A phase I study was done to characterize its safety and pharmacological properties (54). For antibodies like nimotuzumab, the optimal biologic dose is defined as the lowest dose at which there is a saturation of the systemic clearance, based on the assumption that zero-order elimination is associated with the target saturation at the peripheral compartment (54). According to the Phase I pharmacokinetic data, 200 mg was defined as the optimal biologic dose. The investigators concluded that the antibody was very safe at doses from 50 to 400 mg (54). Apart from other molecules in its class, nimotuzumab does not provoke hypomagnesemia, diarrhea, severe cutaneous reactions or thromboembolic events, that are usually induced after the use of EGFR targeting MAbs or STKI (29). Nimotuzumab has been used in at least 7 completed clinical trials in patients bearing advanced SCCHN, concurrently with irradiation or chemo-radiotherapy. This paper summarizes the most important data obtained after adding the antibody to radiotherapy or radio/chemotherapy, according the evolving guidelines in patients with locally disseminated carcinomas.

In a phase I/II clinical trial, 4 individual doses including 50, 100, 200, and 400 mg of nimotuzumab (6 weekly doses), were evaluated (38). Combination therapy consisted of ionizing radiation in doses of 200 cGy for 6 or 7 weeks to complete 6,000–7,200 Gy (38). In general, nimotuzumab was very well-tolerated since no severe or very severe adverse reactions were reported. Most frequent nimotuzumab-related events comprised mild or moderate anorexia, chills, headache, fatigue, and fever (38). Most frequent events attributed to radiotherapy were mucositis, odynophagia, dry mouth, and radiodermitis (38). Authors concluded that there was no exacerbation of the irradiation associated toxicity after using nimotuzumab (38). Overall survival significantly increased with radiotherapy plus nimotuzumab at doses above 200 mg compared to 50 and 100 mg (*p* = 0.03) (38). Median survival of subjects treated with 200 or 400 mg was 44, 30 months and 66.7% of the patients were alive after 3 years (38). It is important to highlight that at the moment of the trial execution, locally advanced SCCHN patients received irradiation alone and not the combination of chemotherapy and radiotherapy, according the guidelines.

In a second single arm study, done at the Princess Margaret Hospital in Canada, nimotuzumab was evaluated together with irradiation. Eligible patients had newly diagnosed stage III/IV SCCHN not amenable to surgery (55). Patients with distant metastases or serious comorbidities were excluded (55). Radiotherapy treatment consisted of 7,000 cGy in 35 fractions without concurrent chemotherapy (55). Nimotuzumab was administered at 100 or 200 mg, for 7 weeks (55). Twenty-four patients received the planned therapy. Most common side effects were grade 1 and 2 nausea, vomiting, headache, and fatigue (55). Of the 12 subjects evaluable for response in each cohort, 10 in the 100 mg group and 11 in the 200 mg group achieved complete or partial response, respectively (55). Authors concluded that nimotuzumab was well-tolerated by most patients and improved the response to radical radiotherapy in patients with SCCHN (55).

## Pharmacodynamic Data

Pharmacodynamics was evaluated in 2 trials where nimotuzumab was used in combination with irradiation in the same population (38, 56). Proliferation was measured through the Ki67 nuclear antigen staining and angiogenesis was estimated using an anti-Von-Willebrand factor antibody. Proliferative activity declined significantly after 3 weekly doses of nimotuzumab (38). Similarly, tumor biopsies showed a remarkable decrease in blood vessels density (38). A second pharmacodynamic study was done in patients with inoperable SCCHN, unfit for concurrent radio and chemotherapy (56). Paired skin and tumor biopsies were obtained before and after the first nimotuzumab administration (56). Immunohistochemistry was used to assess the nimotuzumab impact on the signal transduction cascade on paired skin and tumor biopsies. EGFR expression (regardless the activation status) was not modified by the antibody, either on the skin or tumor biopsies (56). In the skin, p-EGFR (phosphorylated EGFR) in keratinocytes did not decrease after the use of nimotuzumab compared with pre-treatment biopsies (56). On the contrary, nimotuzumab was able to significantly inhibit the HER1 activation (phosphorylation) in the tumor samples (56).

## Vaccinal Effect

Some monoclonal antibodies can exert anti-tumor effect by recruiting effector cells from the innate and adaptive immune system (57, 58).

In addition to the effect in the signal transduction cascade, the capacity of nimotuzumab of increasing the proportion of T-lymphocytes recognizing HER1 (EGFR) peptides *in vivo* was assessed in subjects with SCCHN (59). Peripheral blood mononuclear cells from patients receiving multiple doses of nimotuzumab were cultured with a pool of HER1 peptides. Interferon gamma (IFN γ) producing T lymphocytes were quantified by ELISpot and compared with nimotuzumab naïve patients. There was a significantly higher fraction of IFN γ-secreting T lymphocytes recognizing the EGFR peptides, in subjects with prolonged therapy with nimotuzumab (59). The vaccinal effect mechanism could be attributed to the stimulation of immunogenic apoptosis or the activation NK cells exerting antibody-dependent cell cytotoxicity (ADCC). NK cells trigger dendritic cells activation, that stimulate the HER1-recognizing T lymphocytes in subjects receiving repeated nimotuzumab doses (34, 59). In summary, aside from blocking EGFR or inducing tumor lysis through the NK cells, nimotuzumab induce specific memory T cells against the EGFR, responsible for long-lasting clinical responses.

Furthermore, the percentage of regulatory T cells (Tregs) significantly increased in these SCCHN patients after receiving radio-chemotherapy and nimotuzumab. However, the frequency of Tregs decreased to baseline values with nimotuzumab maintenance (59). Regulatory T cells constitute an important immune-escape mechanism of tumors (60). Authors concluded that nimotuzumab can exert a “vaccinal effect” and circumvent one of the tumors induced immunosuppression ways (59).

## Randomized Studies

The first controlled trial enrolled 106 patients with advanced loco-regional SCCHN, not suitable to receive concurrent chemo-radiotherapy (43). Patients were randomized to receive irradiation plus nimotuzumab or placebo (43). In the experimental group, 70.37% of the enrolled subjects presented side events of any attribution while in the control arm, 57.69% of the patients receiving irradiation and placebo had adverse events (43). Nimotuzumab attributed reactions consisted primarily on grade 1 or 2 fatigue, fever, chills, headache, and appetite loss (43). Radiotherapy associated side reactions in both groups were mucositis, radiodermitis, odynophagia, and dry mouth (43). Authors concluded that nimotuzumab did not increase irradiation toxicity (43).

The trial endpoint was the assessment of response rate in both groups (43). Complete response rate was 59.5% in the antibody plus radiotherapy cohort vs. 34.2% in the control arm. Differences in complete response were statistically significant (43). Median overall survival was 12.50 vs. 9.47 months in the experimental and control groups (43). Median overall survival was lower than the expected for newly diagnosed subjects, provided the poor performance status and comorbidities of the enrolled population, which precluded treatment with concurrent chemo-radiotherapy (43). Differences in survival were significant according Harrington-Fleming, a highly sensitive test to detect differences deferred in time (43).

After the approval of chemo-radiotherapy as the recommended regimen for advanced SCCHN, nimotuzumab was combined with cisplatin-based chemotherapy and irradiation.

In a controlled study done in India leaded by the Kidwai Memorial Institute of Oncology in Bangalore, 92 stage III/IVA patients were randomly allocated to 2 groups: group A (radiation vs. radiation + nimotuzumab) and group B (chemo-radiotherapy vs. chemo-radiotherapy + nimotuzumab). Subjects received 60–66 Gy in combination with 6 weekly antibody doses (61).

Chemotherapy consisted of cisplatin, 50 mg/week for 6 weeks in group B (61). After the 3-agents combination, the most frequent toxicities were mild to moderate asthenia, dizziness, microscopic hematuria, vomiting, fever, chills, itching, skin rash, headache, and hypertension (61). In group A, the objective anti-tumor response was 76% in subjects who received radiation and nimotuzumab vs. 37%, in patients receiving radiotherapy only (61). In group B, the rate of loco-regional control was 100% in patients receiving nimotuzumab plus chemo-radiation vs. 70% in patients treated with chemo-radiotherapy (61).

After long-term follow-up, the 5-year survival rate was 39% with radiotherapy + nimotuzumab vs. 26% for radiotherapy alone (*p* > 0.05) (62). Survival rate was 57% for the chemo-radiotherapy-nimotuzumab group vs. 26% for chemo-radiotherapy (*p* = 0.03), after 5 years (62). The risk of death was 24% lower if patients received radiotherapy + nimotuzumab compared to radiotherapy alone while the death risk was 64% lower with chemo-radiotherapy + nimotuzumab compared to chemo-radiotherapy (62).

Recently, a phase III study designed to assess the impact of nimotuzumab plus cisplatin and irradiation in progression-free survival (PFS) was completed (63). Other goals of the study were the evaluation of disease-free survival, loco-regional control (LRC) and overall survival vs. cisplatin/irradiation. Safety and treatment completion of the regimen were also assessed (63). Five hundred thirty-six (536) subjects were enrolled in the phase III trial done at the Tata Memorial Hospital, in Mumbai, India (63). Selection criteria were age >18 years, confirmation of squamous carcinomas of the oral cavity, oropharynx, hypopharynx, or larynx, stage III–IV, and no distant metastasis. Patients had proper functioning of organs and systems and were fit to receive chemo-radiotherapy (63).

At the time of inclusion, the 2 cohorts were balanced in relation to the key demographic and tumor variables (63). Overall, most subjects showed a good performance status and the primary tumors were mainly from the oropharynx and larynx (63). More than 75% of the patients carried bulky tumors (T3 or T4) and more than 50% of patients had N2 or N3 (52). A large proportion of patients (65%) had stage IVA tumors at the time of recruitment. Remarkably, roughly 70% of the evaluated samples were HPV negative (63).

Patients were allocated to chemotherapy (weekly cisplatin 30 mg/m^2^ dose, 7 weeks) + radiation therapy (total dose of 6,600–7,000 cGy) or the same treatment plus weekly nimotuzumab (200 mg/dose) to complete 7 doses (63). Overall, there was good adherence to chemotherapy and radiation. The median number of cisplatin cycles for both arms was 7 (63). More than 80% of the patients in both cohorts received 7 cycles of cisplatin and <10% of subjects required cisplatin dose reduction (63). More than 75% of the patients received a total cisplatin dose >200 mg/m^2^, comparable with the dose of cisplatin administered in the high-dose chemotherapy schemes (cisplatin 100 mg/m^2^ every 3 weeks) (63). Patients received 7 doses of nimotuzumab (median number of doses), according the planned scheme. No differences in treatment compliance were found, and researchers concluded that the use of nimotuzumab did not affect completion of cisplatin and irradiation (63).

Subjects receiving nimotuzumab together with cisplatin and radiotherapy had a significant and clinically meaningful advantage in PFS: 60.3 months (nimotuzumab group) vs. 21 months (control group) (HR 0.69; *p* = 0.004) (52). The PFS rate at 2-years was 61.8% (nimotuzumab arm) vs. 50.1% (control arm) (63). Subjects receiving an accumulative cisplatin dose ≥200 mg/m^2^ also had a better PFS (HR, 0.73; *p* = 0.036). The PFS HR for patients with oropharyngeal cancers that were p16-negative was 0.54 (63).

The differences in DFS were also significant (HR 0.71; *p* = 0.008) (63). Two-year DFS was 60.2% (nimotuzumab group) vs. 48.5% (control). Nimotuzumab treated subjects also had better response rate. Two-year LRC was 67.5% (nimotuzumab arm) vs. 57.6% (control arm) (HR 0.67; *p* = 0.006) (63).

The adverse events frequency was similar in the 2 groups (63). Most common events were associated with the natural history of advanced head and neck disease and concurrent use of chemo and irradiation (63). Cutaneous rash was more frequent in those subjects receiving nimotuzumab plus chemo-radiation therapy (63). Despite the triple combination, only 17% of patients developed rash of any degree (63). The proportion of patients who developed grade 3–5 events did not differ in both arms of treatment, with the exception of mucositis. Grade 3–5 mucositis was more frequent in the experimental arm (63).

After the marketing approval of nimotuzumab in Cuba, a Phase IV study was conducted in 225 subjects (64). Newly diagnosed patients were treated with nimotuzumab plus irradiation and chemotherapy while unfit subjects for radio or chemotherapy had other treatment modalities (64). Cisplatin was administered every 21 days at a dose of 75–100 mg/m^2^ or weekly (40 mg/m^2^), according each patient performance status or comorbidities (64). One- and two-year PFS for those subjects receiving cisplatin, irradiation plus nimotuzumab was 67.3 and 46.3%, respectively (64). For those treated with radiotherapy and nimotuzumab, 1 and 2-year PFS was 42.9 and 28.6%, respectively (53). Most frequent events related to the antibody were nausea (25%), neutropenia (13.9%), anorexia (19.4%), fever (11.1%), anemia (13.9%), asthenia (16.7%), and vomiting (8.3%). Adverse reactions were grade 1 or 2 and non-serious (64).

## High Dose Chemotherapy vs. Low Dose Chemotherapy for SCCHN

Concurrent irradiation and chemotherapy is the recommended regimen for locally advanced SCCHN patients (65). The current standard protocol of chemo-radiotherapy, involves the use of radiotherapy concurrent with cisplatin bolus dose of 100 mg/m^2^ infused every 21 days (65). Despite the good results with this protocol, treatment-related toxicity remains an important concern. Unacceptable toxicity provokes a delay of the definitive radiotherapy, which, in turn, negatively affects the general therapeutic outcome, especially in elderly patients or in subjects with poor general conditions (65).

In the most recent studies, nimotuzumab was used in combination with low dose cisplatin and radiation (62–64).

There are many recent evidences, supporting the non-inferiority of low cisplatin dose as compared to the high dose regimen. Evidences come from retrospective meta-analysis, systematic reviews or prospective randomized studies (65–72). Mohamed and co-workers did a systematic review that contrasted adverse events and efficacy of once-a-week vs. every 3-weeks cisplatin in stage III/IV SCCHN (66). Authors studied 1,500 trials reported between 1970 and 2015, of which, 39 studies were eligible for the research. Cisplatin weekly doses ranged from 30 to 40 mg/m^2^. Locoregional control was 58 vs. 61% (*p* = 0.7), weekly vs. triweekly (66). Overall survival rate after 2 years was also similar: 74% (once-a-week) vs. 67% (every 3-weeks) (*p* = 0.67) (66). PFS rate at 24 months was 69 vs. 62% (once-a-week vs. every 3-weeks) (*p* = 0.9) (66). Authors concluded that both schemes were equivalent regarding clinical benefit (66).

Szturz et al., made a meta-analysis of 59 studies including more than 5,000 patients (67). For the conventional concurrent chemotherapy and irradiation, the high- and low-dose schemes had comparable survival (67). However, the 3-weekly protocol displayed greater hematology adverse reactions, nausea, vomiting and renal problems, typically cisplatin-related adverse events (67). Treatment compliance was better in the low dose vs. high dose regimen (88 vs. 71%, *p* = 0.0017) (67).

Guan and cols. did a meta-analysis of several trials comparing weekly low-dose to high-cisplatin dose every 3 weeks (68). In the study, which included 779 patients, survival was similar among the two groups. Subjects receiving weekly cisplatin had fewer gastrointestinal adverse effects but greater grade 3–5 mucositis (68).

Jacinto et al., made another meta-analysis of the published papers to evaluate the efficacy of cisplatin once-a-week vs. 3-weekly, in the definitive and adjuvant therapy of SCCHN patients (69). Seven studies were included in the review. Authors concluded that PFS and survival rate at 1 year were similar between the weekly and every 3-weeks arms. The same results were confirmed after 5 years follow-up (69).

In another study, Bauml and cols, examined the outcome of patients with locally advanced SCCHN treated with concurrent irradiation and cisplatin at high-dose (HDC) or low-dose (LDC), using Veterans Affairs data across the United States (70). The research involved 2,901 subjects. After correcting for performance status, high dose did not improve overall survival (HR 0.94) (70). However, HDC groups had augmented risk of renal failure, leukopenia, and hearing damage (70).

Apart from these retrospective meta-analyses, Lee et al. did a prospective study including 220 South-Korean patients, of which 65 received cisplatin at a dose of 100 mg/m^2^ every 21 days while 155 subjects got cisplatin weekly doses of 30–40 mg/m^2^ (60). In this controlled study, median PFS of the weekly group was not different to the high dose group (*p* = 0.81) (71). The 3-year overall survival and PFS were similar between the groups (71).

Helfenstein and collaborators in Switzerland conducted a prospective clinical trial where 314 patients were treated with weekly or tree-weekly cisplatin (72). Mean total dose was 200 mg/m^2^ for subjects in the tree-weekly regimen and 160 mg/m^2^ in the weekly scheme. A cumulative cisplatin dose ≥200 mg/m^2^ did not translate into a survival benefit (72).

We conclude that alternative regimens with lower cisplatin dose reduce toxicity, permit good treatment completion and preserve antitumor effect. This was precisely the regimen selected for the recent nimotuzumab-controlled trial.

## Chemo-Radiotherapy Combined With Other Anti-EGFR Monoclonal Antibodies

Cetuximab had shown efficacy in the treatment of recurrent or metastatic head and neck tumors (73). However, for newly diagnosed advanced patients with PS of 0–1, the recommended treatment is concurrent systemic therapy with cisplatin or carboplatin and radiotherapy (74). Cetuximab can also be recommended in combination with radiotherapy for the systemic treatment of locally advanced head and neck cancer (74). In the Bonner trial, 424 patients with locally advanced stage III/IV squamous cell carcinomas of the hypopharynx, oropharynx and larynx received definitive RT with or without cetuximab (75). Median survival time was 49 vs. 29.3 months (75). The trial included mainly patients with oropharyngeal tumors (60%) (75). In a secondary analysis of the trial including only patients with larynx or hypopharynx tumors, there were no differences in laryngeal preservation, laryngectomy free survival, and overall survival (65). In general, the combination of cetuximab and radiotherapy is prescribed to patients unfit to receive cisplatin and radiotherapy, provided that several trials, mainly done in the scenario of HPV positive oropharynx cancer, demonstrated that cetuximab/RT was inferior to cisplatin/RT (76–78).

Two clinical trials with the anti-HER1 MAbs cetuximab and panitumumab, failed in augmenting the effect of radiotherapy plus high-dose cisplatin in SCCHN patients with stage III/IV tumors (79, 80).

RTOG 0522 was a Phase III study that test the hypothesis that adding cetuximab to radiotherapy-cisplatin (100 mg/m^2^) improves PFS (79). Locally advanced SCCHN patients were treated with cisplatin/irradiation with or without cetuximab (79). The addition of cetuximab did not increase PFS, overall survival, loco-regional control, or distant metastasis (79). The regimen caused more serious adverse effects, which had a negative impact on the radiotherapy compliance, neutralizing any potential benefit in tumor control (79). There were more treatment related deaths and grade 3–4 events including rash, fatigue, anorexia, hypokalemia, as well as more acute radiation mucositis in the cetuximab cohort (79). Authors concluded that adding cetuximab to the standard treatment of radiation-cisplatin did not improve the clinical benefit, had worse treatment compliance and greater toxicity (79). Table 2 compares nimotuzumab and cetuximab phase III clinical trials in combination with chemotherapy and radiotherapy for the treatment of locally advanced head and neck cancer.

**Table 2:** Comparison between nimotuzumab and cetuximab Phase III clinical trials in combination with chemotherapy and radiotherapy for the treatment of locally advanced head and neck cancer.

	**Nimotuzumab SCCHN/2010**	**Cetuximab RTOG0522**
Number of patients	536 newly diagnosed, stage III or IV locally advanced squamous cell carcinomas from the oropharynx, larynx, hypopharynx, or oral cavity	891 stage III or IV (T2N2-3M0 or T3-4, any N, M0) squamous cell carcinoma of the oropharynx, hypopharynx, or larynx
Primary endpoint	Progression free survival	Progression free survival
P16 positivity	11.3%	73.2%
Treatment schedule	Cisplatin dose: 30 mg/m^2^, weekly RT dose: 70 Gy Nimotuzumab dose: 200 mg, weekly for 7 weeks	Cisplatin dose: 100 mg/m^2^, on days 1 and 22 of RT. RT dose: 70–72 Gy. Cetuximab dose: 400 mg/m^2^ (induction), then 250 mg/m^2^ weekly for 7 weeks.
Treatment compliance	No differences in radiotherapy interruption between arms. Radiotherapy interruptions as a result of toxicity was 4.5% in the nimotuzumab arm vs. 3.7% in the control arm.	Radiotherapy interruption as a result of toxicity was significantly higher in the cetuximab vs. control arm (26.9 vs. 15.1%) *p* < 0.001).
MAB doses	84.3% received 7 or more weekly nimotuzumab doses	73.6% received 6 or more weekly cetuximab doses
Efficacy	Significant improvement in PFS, locoregional control, and disease-free survival with nimotuzumab. Trend toward improved survival.	No significant differences between arms in PFS, overall survival, locoregional failure, or distant metastases.
Safety	Grade 3–5 adverse events were similar between the 2 arms, except for a higher incidence of mucositis in the nimotuzumab vs. control arm (66.7 vs. 55.8%; *p* = 0.01). No differences in the treatment related deaths	Cetuximab arm had significantly higher rates of grade 3–4 skin reactions, radiation mucositis, fatigue, anorexia, and hypokalemia up to 90 days from the start of therapy. More treatment-related grade 5 adverse events in the cetuximab arm (*p* = 0.05).

In Concert 1, 153 subjects with stage III, IVa, or IVb SCCHN were allocated to chemoradiotherapy or panitumumab plus chemoradiotherapy (80). The main variable of the study was the 2-year LRC while secondary endpoints were PFS and overall survival (80). No differences were found in loco-regional control, PFS and survival between the 2 arms (80). Severe and very severe adverse reactions (dysphagia, mucosal inflammation, and radiodermitis) were more frequent in the panitumumab group. Authors did not recommend adding panitumumab to irradiation and cisplatin for treating SCCHN patients (80).

## Concluding Remarks

Head and neck squamous cancer encompasses a great variety of tumors originating in the oral cavity, oropharynx, hypopharynx, lip, nasopharynx, or larynx. A large proportion of patients with SCCHN debuts with a tumor at locally advanced stage (11). These patients require a multidisciplinary care. In this scenario, chemo-radiotherapy is the standard approach (9, 12).

EGFR expression has been linked to radio-resistance of head and neck tumors (81, 82). Consequently, molecular targeting of EGFR for radio-sensitization stays as a very appealing strategy.

Nimotuzumab is a genetically engineered MAb with an intermediate affinity against the EGFR. Nimotuzumab is the safest antibody in its class since it selectively targets those tumor cells with a high expression of the receptor, while not binding to normal tissues. This is precisely the case of SCCHN, that exhibit a very high receptor expression. The distinctive effect in tumor and normal tissues is demonstrated by the low incidence of skin rash together with good antitumor activity. A different pharmacodynamic effect was confirmed when comparing serial tumor and skin biopsies: nimotuzumab was able to abrogate signal transduction in the tumor but not in the keratinocytes.

Besides inhibiting the signal transduction cascade, nimotuzumab behaves as an active immunotherapy, inducing EGFR specific T cells and reducing the Tregs. These immunomodulatory properties can explain the antibody long-lasting effect.

This review summarizes the results of seven single-arm or randomized clinical trials, where nimotuzumab was combined with radiotherapy or radiation and cisplatin. Collectively, these data support the effectiveness of HER1 inhibition with nimotuzumab in the curative management of stage III/IV tumors from the oral cavity, oropharynx, hypopharynx, and larynx.

## Author Contributions

TC and NI participated in the design of the clinical trials done in Cuba. ZM characterized the mechanism of action of nimotuzumab in terms of inducing cellular immunity. BM participated as a clinical researcher in some of the studies, as a KOL from the Cuban Institute of Oncology and Radiobiology. All authors reviewed the published literature on nimotuzumab and the final manuscript.

## Conflict of Interest

The authors declare that the clinical trials described here-in received funding from the Center of Molecular Immunology, Cuba, the Cuban Ministry of Public Health, YMBiosciences (Canada), Biocon LTD (India), and the TATA Memorial Center Research Administration Council (India). YMBiosciences, Biocon LTD and the TATA Memorial Center Research Administration Council were not involved in this paper design, collection, analysis, interpretation of data, writing of this article or the decision to submit it for publication. Three authors (TC, ZM, NI) currently work or have worked for the Center of Molecular Immunology, the institution that owns the patent and manufactures nimotuzumab. NI was employed later by Biocubafarma. The remaining author declares that the research was conducted in the absence of any other commercial or financial relationships that could be construed as a potential conflict of interest.
